# Exploring HIV/AIDS investigator perceptions of equity within research partnerships between low-and middle-income and high-income countries: a pilot survey

**DOI:** 10.1186/s12961-023-00977-9

**Published:** 2023-05-01

**Authors:** Chelsea E. Modlin, Edward Nelson Kankaka, Larry W. Chang, Nelson K. Sewankambo, Joseph Ali

**Affiliations:** 1 Division of Infectious Diseases, Johns Hopkins University School of Medicine, Baltimore, MD USA; 2 Johns Hopkins Berman Institute for Bioethics, Baltimore, MD USA; 3 Rakai Health Sciences Program, Rakai, Uganda; 4 Department of International Health, Johns Hopkins Bloomberg School of Public Health, Baltimore, MD USA; 5 Department of Medicine, Makerere University College of Health Sciences, Kampala, Uganda

**Keywords:** Research equity evaluation, International research partnerships, Global health, HIV/AIDS

## Abstract

**Background:**

Recommendations for research partnerships between low- and middle-income countries (LMICs) and high-income countries (HICs) stress the importance of equity within the collaboration. However, there is limited knowledge of the practical challenges and successes involved in establishing equitable research practices. This study describes the results of a pilot survey assessing key issues on LMIC/HIC partnership equity within HIV/AIDS research collaborations and compares perspectives of these issues between LMIC- and HIC-based investigators.

**Methods:**

Survey participants were selected using clustered, random sampling and snowball sampling. Responses were compared between LMIC and HIC respondents using standard descriptive statistics. Qualitative respondent feedback was analyzed using a combination of exploratory and confirmatory thematic analysis.

**Results:**

The majority of categories within four themes (research interests and resources; leadership, trust, and communication; cultural and ethical competence; representation and benefits) demonstrated relative consensus between LMIC and HIC respondents except for ‘lack of trust within the partnership’ which was rated as a more pronounced challenge by LMIC respondents. However, subcategories within some of the themes had significant differences between respondent groups including: equitable setting of the research agenda, compromise within a partnership, the role of regulatory bodies in monitoring partnerships for equity, and post-study access to research technology.

**Conclusions:**

These efforts serve as a proof-of-concept survey characterizing contemporary issues around international research partnership equity. The frequency and severity of specific equity issues can be assessed, highlighting similarities versus differences in experiences between LMIC and HIC partners as potential targets for further discussion and evaluation.

**Supplementary Information:**

The online version contains supplementary material available at 10.1186/s12961-023-00977-9.

## Background

Collaborative partnerships between researchers in low- and middle-income countries (LMICs)^1^ and those in high-income countries (HICs) are a common model for global health research conducted in LMICs [1, 2]. International research can take many forms, including projects isolated to one study or a brief period of time. While attention to equity in these shorter-term arrangements is still necessary, the term ‘partnership’ is referred here to mean longitudinal collaborative efforts between international research teams that share mutual interests or scientific objectives. These partnerships often function as pragmatic solutions to address prominent health research needs in LMICs, which face underlying structural and economic challenges that can otherwise slow health research progress. By combining expertise and resources between LMIC and HIC partners, these partnerships could potentially produce valid, locally-relevant research that contributes to scientific knowledge production, translates to important health outcomes, and strengthens efforts to build and sustain local research infrastructure.

However, international research partnerships between LMICs and HICs are not without significant problems given the contexts of power imbalances and resource inequities in global health. These include, but are not limited to, differences in research experience, scientific leadership, research topic interests, institutional support, financial transparency, material resources [3], representation in research outputs [4, 5], and post-study access to data and technology [6, 7]. Asymmetries between global health research partners are further complicated by the direct and indirect impact of residual colonial influences that, in many instances, permeate contemporary academic global health discourses and infrastructure [8, 9]. These complicated factors surrounding international research partnerships present risks to equitable partnership practice and outcomes, and make clear that further efforts are needed to identify and address partnership inequities. This includes developing an accurate account of successes as well as barriers that have inhibited both LMIC and HIC collaborators from advancing equity in their joint pursuits.

Equitable efforts and outcomes within research partnerships are prioritized as one of the United Nations’ Sustainable Development Goals [2] and are considered essential for the conduct of ethical partnership-based research in LMICs [10, 11] with a normative foundation based on theories of social and global justice [12]. While there are efforts to promote equitable practice within international research collaborations, notably the Research Fairness Initiative (RFI) developed by the Council on Health Research for Development [13], systematic efforts such as building relevant equity metrics and assessing partnership-specific interventions have been sparse. Within the academic literature, guidelines and recommendations are approaching consensus [14–16] on the principles and key determinates of what constitutes research partnership equity. However, formal assessments of pragmatic equity challenges have largely been restricted to commentaries, opinions, qualitative investigations [17], and discussions around bibliographic trends [4, 18–20]. More comprehensive understanding of how guidelines translate into practice and defining the role of partnership evaluation tools are needed to fully capture the scope of these issues and move the field forward.

Combined with the growing globalization of medical research, HIV/AIDS has prompted an extraordinary acceleration and evolution of paradigms within global public health and clinical research [21]. With its prominence as a major contributor to the global health landscape, research programs focusing on HIV/AIDS frequently suffer from the same complex structural imbalances that have been reported in global health research more generally [22]. In this report, we summarize efforts to develop and pilot a survey about research partnership equity among investigators who conduct HIV/AIDS research in LMICs. The design allows for a direct contrast of perspectives between LMIC- and HIC-based researchers and the identification of topics that share similar versus diverging opinions. Because of the diversity of interests, objectives, and processes that vary between partnerships, this survey also offers pragmatic prioritization of topics among the many equity-related considerations so that those most likely to benefit the specific needs of an individual partnership are highlighted. The main objective of the pilot was to obtain preliminary results to refine future content so that subsequent survey iterations are better able to describe and compare stakeholder-identified practices that exemplify or prevent the promotion and practice of equity within international research partnerships between LMICs and HICs. Using HIV/AIDS research as a case study, this survey is part of a larger research program that ultimately aims to develop and implement pragmatic equity-specific evaluation tools and metrics for global health research partnerships.

## Methods

### Survey

The survey was developed through a targeted narrative review of the literature (for examples please see references [14, 23–25]) and recommendations for research partnership equity (for examples please see references [13, 26–28]). This review was conducted by author CEM using PubMed/MEDLINE, Scopus, and Google Scholar databases using keyword searches for ‘equity’ OR ‘fairness’ AND ‘international research partnerships’ OR ‘global research partnerships’ ‘OR ‘transnational research partnerships’ OR ‘North–South partnerships.’ Bibliographies of reviewed papers were cross-referenced with the literature search findings and relevant articles were retrieved if not included in the original search. Equity-related categories were preliminarily extracted from research partnership equity frameworks to create a text-by-theme matrix where subsequent articles from the literature were reviewed for categories within the matrix and also underwent evaluation for novel categories. The matrix categories were iteratively revised and grouped in a constant comparative fashion. Categories and thematic groups were discussed throughout the review with authors ENK, LWC, and JA to minimize bias. From this analysis and the final thematic matrix four themes emerged: (1) Research interests, agenda, and resources (3 categories, 14 subcategories), (2) Leadership, trust and communication (5 categories, 16 subcategories), (3) Cultural competence and good research practice (3 categories, 17 subcategories), and (4) Research representation and benefits (5 categories, 23 subcategories). Questions were initially revised based on feedback from six key informants with expertise in international HIV/AIDS research (*n* = 3), global health ethics (*n* = 2), and qualitative and quantitative survey design (*n* = 1) to generate the content and structure of the pilot survey.

The pilot survey was designed to first ask respondents to roughly estimate (“not at all”, “a little bit”, “a lot”, or “a great deal”) how much categories within each theme contributed to equity barriers or facilitators they had experienced or witnessed. To improve the efficiency and usability of the survey given a large number of themes/categories of interest, skip patterns were created that allowed for more specific questioning to only be applied for categories respondents selected as substantial barriers to equity promotion and practice. For example, when asked about how much ‘disproportionate financial and material resources between partners’ affected partnership equity, if a respondent selected ‘a great deal’ or ‘a lot’, they would be directed to a series of additional 5-point Likert-type questions (ranging from ‘strongly disagree’ to ‘strongly agree’) about specific concepts within the category of material and financial resources. If a respondent answered ‘a little bit’ or ‘not at all’, they would be directed to the next theme. Space for open-ended feedback on survey items was provided within each page as well as summative feedback requested at the end of the survey. Data were collected using Qualtrics XM survey management software.

### Participants

Pilot survey participants were selected using clustered, random sampling and snowball sampling of investigators with experience conducting HIV/AIDS-related research in LMIC settings. The investigator cohort based in the United States was identified by random sampling of faculty from two large academic institutions, Johns Hopkins University in Baltimore, Maryland, and Emory University in Atlanta, Georgia. HIV/AIDS investigators based in LMICs were identified using random sampling from the Johns Hopkins Center for HIV/AIDS Research (CFAR) email listserv. Because there was no specific singular outcome of interest and limited ability to estimate the total population size, the target enrollment of 22 respondents was calculated based on pilot study sample size estimation [28] assuming that if a problem exists with a 10% probability for a study participant, the problem will be identified with 90% confidence. Recruitment and survey completion took place between May 1, 2022, and August 15, 2022. After stratifying by LMIC versus HIC affiliations to ensure equal representation from both settings, in random order, each investigator was sent an email with an individualized link and request to participate until the minimum number of respondents was surpassed. Information about the study was provided on the first page of the survey and respondents were required to consent to participate prior to being directed to the survey content. Respondents who completed at least one question on the survey were provided with a $20 USD electronic gift card. One survey reminder was sent 7–10 days after the initial invite. This study protocol was determined as exempt from review by the Johns Hopkins Medicine Institutional Review Board.

### Analysis

Microsoft Excel was used for de-identified data compilation and basic descriptive statistics. Each response was converted to a quantitative value (not at all = 1, a little bit = 2, etc. for categories; strongly disagree = 1, disagree = 2, etc. for subcategories) to generate standard statistical values. Unequal variances t-test was used to compare means between LMIC and HIC respondents.

Responses to open-ended feedback questions were analyzed qualitatively using NVivo 1.0 (QRS International, released 2020). A combination of exploratory and confirmatory thematic analysis was used, the latter of which used the pre-structured themes and categories as codes. Exploratory data analysis was used to identify new themes and generate categories and codes for constructive feedback. Thematic coding was done independently by two coders and discrepancies were resolved by consensus. The dataset supporting the conclusions of this article is included within the article and its additional files (Additional file 1).

## Results

### Participation and demographics

A flow sheet describing recruitment and participation can be seen in Additional file 2. There were completion rates of 18% (*n* = 14) and 33% (*n* = 13) for LMIC and HIC investigators, respectively. The median time it took participants to complete the survey was 16 min. As depicted in Additional file 3, there was relative diversity within both groups in terms of age, gender, career stage, and area of HIV/AIDS research. Most respondents from HICs identified as Principal Investigators and Co-Investigators, whereas investigators from LMICs were more varied in their professional roles.

### Survey content

**Fig. 1:**
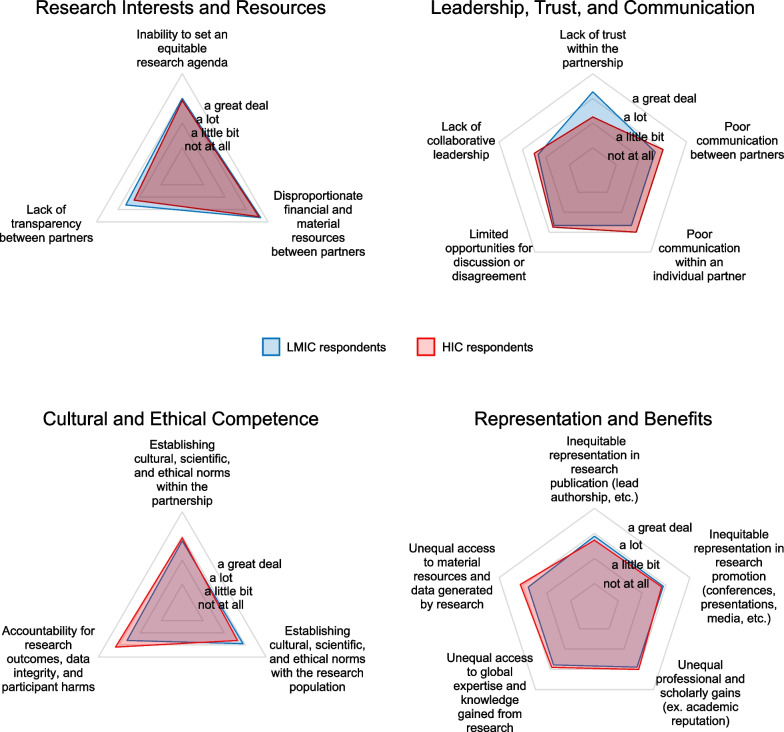
Spider plot of comparative means between LMIC (blue) and HIC (red) survey respondents. Categories within each theme are measured as the degree to which each category contributes to a lack of equitable practice within international research partnerships

The majority of the four major themes and their respective categories (Fig. 1) demonstrated relative consensus between LMIC and HIC respondents except for a category within “leadership, trust and communication.” More specifically, ‘lack of trust within the partnership’ was rated as a more pronounced barrier to partnership equity by LMIC respondents than by HIC respondents. Of all categories, ‘disproportionate financial and material resources between partners’ was the highest-scoring equity barrier while ‘lack of collaborative leadership’ and ‘lack of transparency between partners’ were reported as less significant barriers.

Categories and subcategories within each theme are shown in Tables 1, 2, 3 and 4. Note that the category headings listed in Tables 1, 2, 3 and 4 are the same categories displayed in Fig. 1 and *p*-values reflect the differences depicted in Fig. 1. There were varying degrees of differences between the means of each group with some reaching statistical significance (*p*-value < 0.05) between the means of LMIC versus HIC respondents.

**Table 1.**
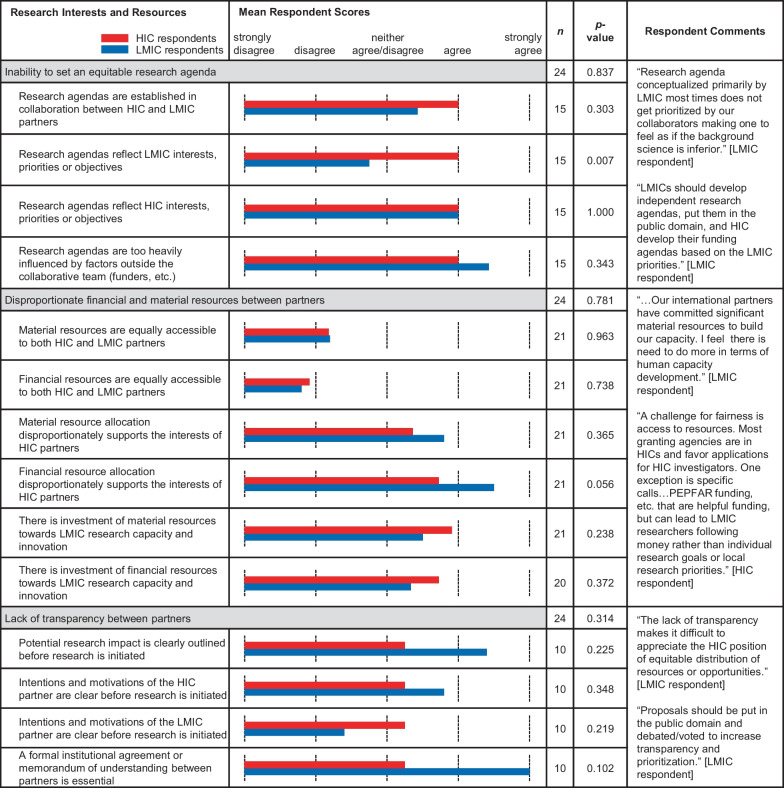
Comparative means within subcategories of ‘Research Interests and Resources’. Bar graph of the means between LMIC (blue) and HIC (red) survey respondents

**Table 2.**
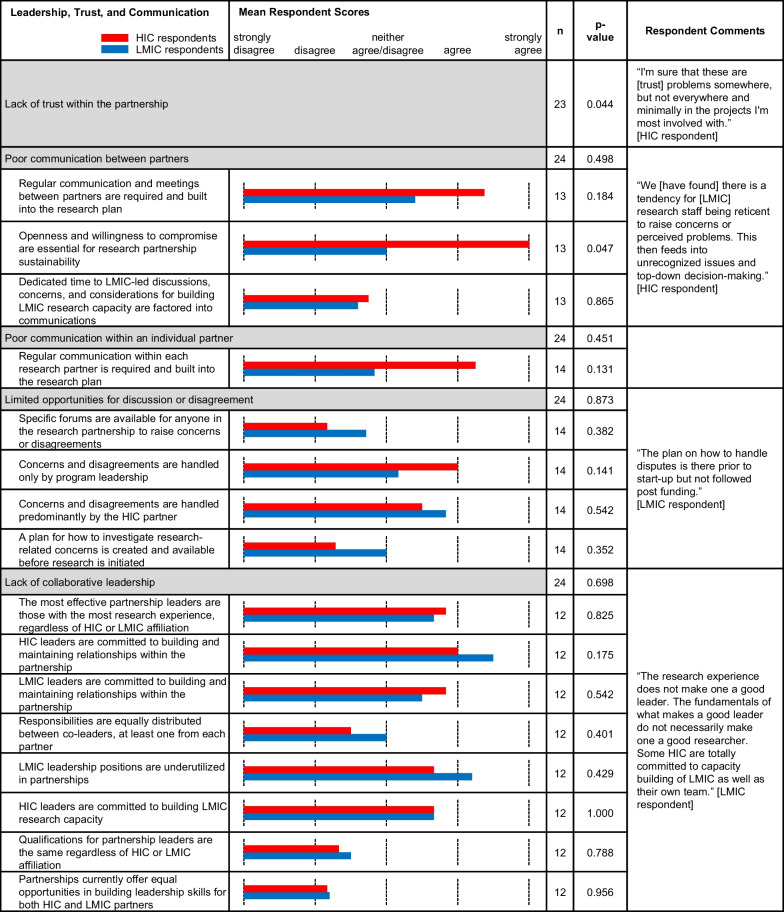
Comparative means within subcategories of ‘Leadership, Trust and Communication’. Bar graph of the means between LMIC (blue) and HIC (red) survey respondents

**Table 3.**
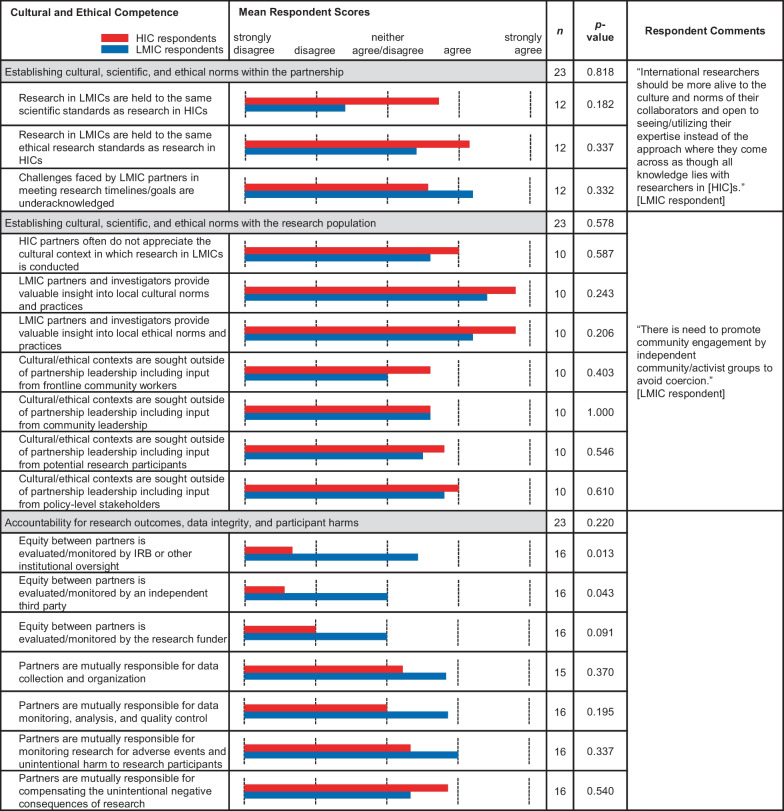
Comparative means within subcategories of ‘Cultural and Ethical Competence’. Bar graph of the means between LMIC (blue) and HIC (red) survey respondents

**Table 4.**
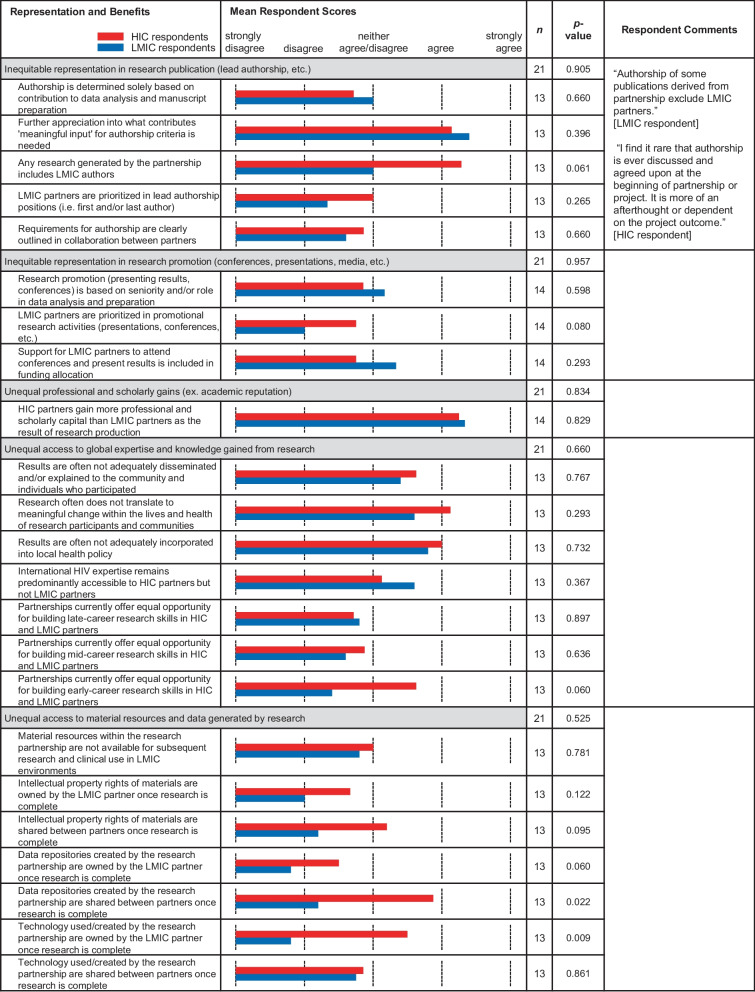
Comparative means within subcategories of ‘Representation and Benefits’. Bar graph of the means between LMIC (blue) and HIC (red) survey respondents

Respondents frequently used the open-ended questions embedded within each theme and category to elaborate on their answers to a particular category or sub-category or to make a more nuanced point. There were only two suggestions of content to potentially include in future iterations of the survey: (1) navigating political corruption external to the partnership and (2) personal accountability and integrity of investigators.

### Survey revision

Exploratory thematic analysis of respondent feedback about the survey generated the following codes: specificity, clarification and definitions, and exploring relationships between themes and categories. Many respondents indicated that further elaboration on the specificity of the survey questions is needed, questioning if the prompts should be answered based on their own experiences versus general perceptions of these issues within the field as a whole. This is particularly relevant given the intention to develop this survey into a partnership-specific evaluation tool which speaks to the need for clarifying that responses should reflect personal experiences. Some respondents requested definitions of terms that were used. This was most often for a specific subcategory, for example, one respondent requested a definition for “research agenda,” and another for what was meant by “mutual responsibility.” One respondent requested clarification of what was meant by “financial resources” and suggested separating this into “institutional resources” and “grant funding”. Two respondents also questioned broader definitions for what was meant by the term “equity” and enquired if this is synonymous with equality. Several respondents suggested an additional survey component where they could rank the categories within a theme against one another in terms of importance or frequency.

## Discussion

This pilot survey is a proof-of-concept attempt at describing and comparing perspectives on international research partnership equity among HIV/AIDS investigators from LMICs and HICs. Our findings demonstrate that a broad range of thematically-organized categories and subcategories can be used to analyze stakeholder opinions. Nevertheless, there are some limitations and lessons learned in the creation and applicability of this survey that will help inform future iterations.

The need for systematic evaluation of equity-related goals and outcomes within international research partnerships is evident. Guidelines for global health research partnerships in LMICs emphasize the need for equity between LMIC and HIC research partners [10, 15, 29]. Yet there is limited existing empirical research to substantiate criteria or identify meaningful outcome metrics for defining, evaluating, and monitoring research partnership equity. The RFI provides a subscription-based reporting platform for high-level systems like academic institutions and governments to reflect on their practices around partnership equity. This is then summarized in an open-ended, 45-item report. While comprehensive, the RFI report is focused on analyzing current program policies and establishing a commitment to equitable practice. In comparison, our survey, while still in its early stages, is more focused on a quantitative design that demonstrates surface-level comparisons between stakeholder-driven priorities and is designed to be used at the individual partnership level to highlight partnership-specific equity interests and practices. We hypothesize that responses demonstrating concordance between research partners may reflect larger systemic factors that influence equity that may not be in the direct control of a specific partnership. However, responses demonstrating discordance between partners highlight a potential target area if a partnership is interested in promoting more equitable practices. For example, a positive response to ‘lack of access to financial resources for the LMIC partner’ may reference the fact that global health funding is frequently funneled through HIC institutions or partners which may lay outside the immediate field of influence for a specific partnership. However, this response could also reference how and where funding is allocated within the partnership which is potentially more feasible for evaluation, discussion, and change to promote more equitable financial distribution. This distinction requires a closer look at the details and nuances that would be best derived from in-depth qualitative follow-up and also trialing the survey within a specific partnership, both of which are planned next steps. Our survey does share some thematic overlap in content with the Equity Tool for Valuing Global Health Partnerships (EQT) [30]. However, the EQT places more emphasis on the role of an individual within a research partnership. Its goal is not to serve as a framework for partnership evaluation, but rather a tool to generate meaningful conversations around equity-related topics.

To date, partnership equity guidelines have not had a clear impact on systematically improving equity within research partnerships [13] although investigations into how to measure these outcomes are ongoing. Further evaluation into pragmatic metrics for equitable partnerships is needed. While still requiring revision and validation within international research partnerships, our survey offers a preliminary assessment tool to capture various issues a partnership may be experiencing and areas where further discussion is needed, such as significant discrepancies between LMIC and HIC research collaborators. It also offers the option of providing a mechanism of comparing pre- and post-responses for any intervention targeting the promotion of research partnership equity, such as the RFI report, or as a mechanism of iterative evaluation of partnerships at specific intervals of time.

Respondents found the survey comprehensive with sparse feedback that the topics and terminology within the survey were unknown or unfamiliar, such as equity vs. equality or definitions for terms like “the research agenda.” This issue can be rectified by the addition of embedded definitions or examples of concepts and themes that are less well-understood. The inclusive scope of this survey content comes at the cost of the length of time it takes a respondent to complete with one respondent suggesting a more streamlined survey interface. Optimal survey formatting and phrasing of the categories and subcategories will be revisited in its revision to try and help address this limitation. This survey is, by no means, adequate to fully capture and describe pluralistic equity-related issues within a research partnership. These issues can range from interpersonal to the microenvironment to the surrounding macrosystems or geopolitical environment of the partnership, some of which are more readily addressable or quantifiable than others, but all are important to acknowledge. Findings should be followed up with in-depth qualitative evaluation such as stakeholder interviews and discussions to generate a more nuanced and partnership-specific understanding of the issues.

As a pilot study, there are several limitations to consider. The survey was developed based on a literature review conducted by one person without a second independent coder which introduces a risk of bias in the selection of themes and categories within the original survey. Future iterations based on the rapidly growing literature on research partnership equity will feature simultaneous review and consensus by at least two independent coders. Limitations also include a small sample size, reflective of two HIC-based institutions, and the sample was targeted to be representative of a prospective survey cohort within active international HIV/AIDS research partnerships. The response rates were low compared to other published surveys distributed to academic medical professionals [31] although half of LMIC respondents did not open the survey email, suggesting some requests may have been automatically routed to junk mail folder or not seen. The next revised administration of this survey among a larger population will include an evaluation for non-response bias. Skip patterns were used to minimize time spent answering questions less relevant to unique respondents, but there is a tradeoff that subcategories within these sections that the respondent may consider significant were overlooked. This was not reflected in responses to the questions asking about topics not covered but remains a possibility. There was a prominence of principal investigators among these respondents, likely related to the recruitment strategy resulting in a sampling bias. At the same time, senior investigators are most likely to have experience reflecting on issues of research partnership equity. Some degree of over-representation may assist with substantiating specific claims, especially if applying this survey within a larger sample size, but verification in other groups of research staff is still needed. With the globalization of HIV/AIDS research and the mobility of investigators, the grouping of respondents as LMIC or HIC is arguably somewhat arbitrary. Future iterations of the survey may ask respondents to self-identify which of these categories they fall into, with an ‘other’ category to explain if this binary description does not accurately represent their background.

Following revisions based on pilot respondent feedback, next steps include validating within an international research partnership and combining survey results with follow-up participant interviews. We hope this line of research will contribute to efforts to characterize contemporary issues around partnership equity, highlight differences and similarities of experience between LMIC and HIC partners as a priority for discussions, and begin to identify concrete, stakeholder-derived metrics and guidance for international research partnerships to strive towards more equitable outcomes.

## Conclusions

Equity between LMIC and HIC research partners within international research partnerships is an essential component of ethical research in LMICs. While efforts to date are starting to acknowledge and converge on the diverse array of themes that fall under the umbrella of research partnership equity, formal mechanisms for identifying practices that facilitate or inhibit equity are limited. We describe a preliminary pilot assessment of a survey designed to identify the frequency and severity of equity-related considerations. This survey also highlights similarities versus differences in experiences between LMIC and HIC partners as potential priority targets for further partnership-level evaluation and intervention.

## Supplementary Information

Additional file 1. Raw data.

Additional file 2. Flowsheet of respondent recruitment and participation. LMIC low- and middle-income country, HIC high-income country.

Additional file 3.Survey participant demographics. Completion was not required and not all participants completed the demographics section of the survey, resulting in different response numbers between the demographic information collected and overall survey responses.

## Data Availability

All data generated or analyzed during this study are included in this published article and its supplementary information files.

## Abbreviations

AIDS: Acquired Immunodeficiency Syndrome
CFAR: Center for AIDS Research
EQT: Equity tool for valuing global health partnerships
HIC: High-income country
HIV: Human immunodeficiency virus
LMIC: Low- and middle-income country
RFI: Research fairness initiative

## Footnotes

1 The term ‘LMIC’ is used in reference to countries that fall under World Bank criteria for low- and -middle income countries as defined by gross national income per capita. This terminology is often used in the academic literature but is an imperfect descriptor that fails to capture important distinctions and variability between countries that make them independent world entities. By using this term, we find it prudent to be clear that the experiences of LMICs with HIV research and research partnerships are not monolithic.
