# Supplementary material for: Exploring HIV/AIDS investigator perceptions of equity within research partnerships between low-and middle-income and high-income countries: a pilot survey
Source: Health Res Policy Syst. 2023 May 1;21:32. doi: 10.1186/s12961-023-00977-9 (PMC10152781; doi:10.1186/s12961-023-00977-9)
Supplement: Supplementary file 2 — Additional file 2. Flowsheet of respondent recruitment and participation. LMIC low- and middle-income country, HIC high-income country. [file 12961_2023_977_MOESM2_ESM.pptx]

## Slide 1
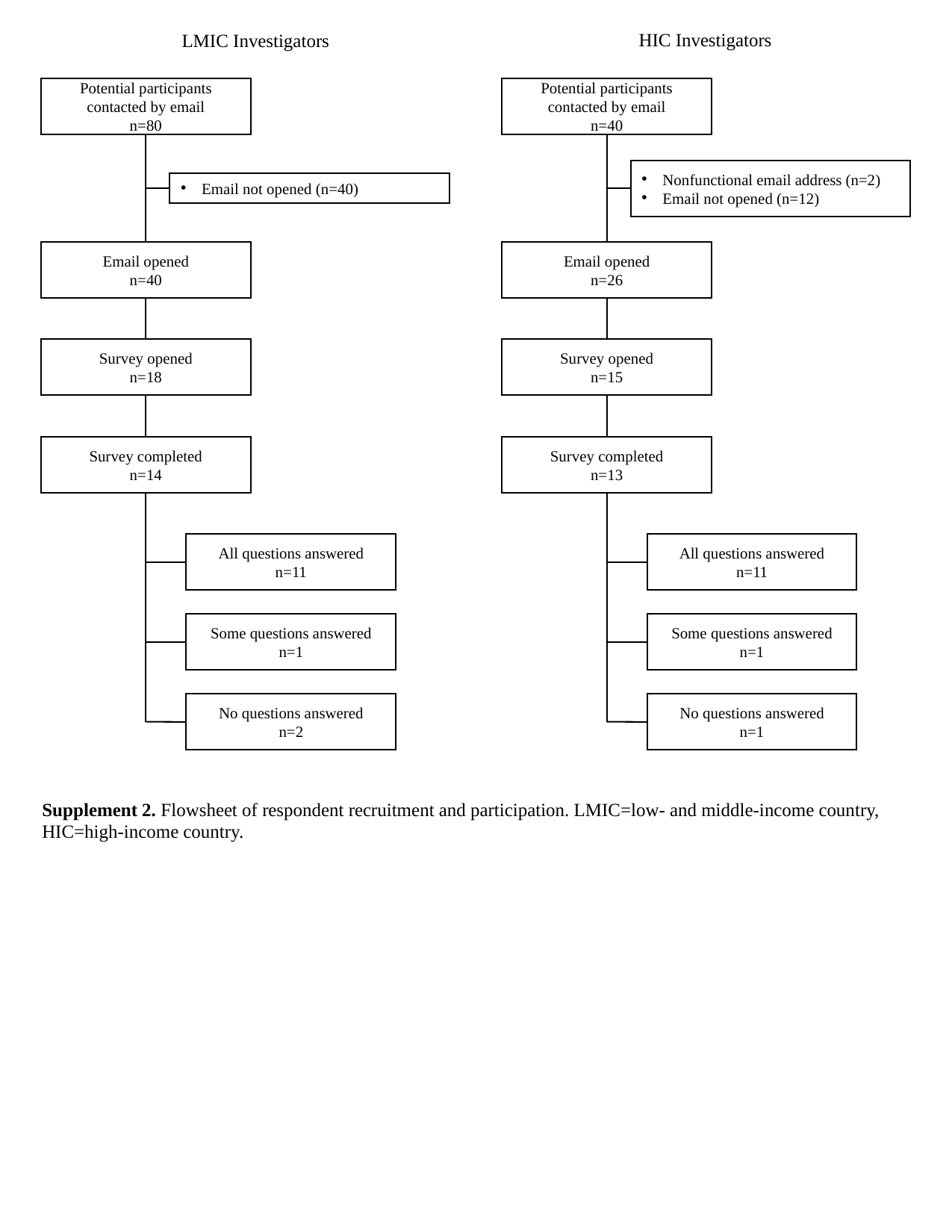

HIC Investigators
LMIC Investigators
Potential participants contacted by email
n=80
Potential participants contacted by email
n=40
Nonfunctional email address (n=2)
Email not opened (n=12)
Email not opened (n=40)
Email opened
n=40
Email opened
n=26
Survey opened
n=18
Survey opened
n=15
Survey completed
n=14
Survey completed
n=13
All questions answered
n=11
All questions answered
n=11
Some questions answered
n=1
Some questions answered
n=1
No questions answered
n=2
No questions answered
n=1
Supplement 2. Flowsheet of respondent recruitment and participation. LMIC=low- and middle-income country, HIC=high-income country.
