# Supplementary material for: Exploring HIV/AIDS investigator perceptions of equity within research partnerships between low-and middle-income and high-income countries: a pilot survey
Source: Health Res Policy Syst. 2023 May 1;21:32. doi: 10.1186/s12961-023-00977-9 (PMC10152781; doi:10.1186/s12961-023-00977-9)
Supplement: Supplementary file 3 — Additional file 3.Survey participant demographics. Completion was not required and not all participants completed the demographics section of the survey, resulting in different response numbers between the demographic information collected and overall survey responses. [file 12961_2023_977_MOESM3_ESM.docx]

**Supplement 3.** **Survey participant demographics.**

| **Demographic** | **LMIC Participants**  (n=9) | **HIC Participants**  (n=12) |
| --- | --- | --- |
| Age | | |
| 30-39 | 1 | 1 |
| 40-49 | 6 | 5 |
| 50-59 | 1 | 5 |
| 60+ | 1 | 1 |
| Gender | | |
| Female | 3 | 4 |
| Male | 5 | 8 |
| Non-Binary | 1 | 0 |
| Racial/Ethnic Identity | | |
| African American | 1 | 0 |
| Black | 6 | 0 |
| Caucasian | 0 | 8 |
| Asian | 1 | 3 |
| LatinX | 0 | 2 |
| Pacific Islander | 0 | 1 |
| Other | 2 | 0 |
| Education | | |
| Doctorate | 3 | 11 |
| Master’s* | 4 | 1 |
| College/University | 1 | 0 |
| Secondary School | 1 | 0 |
| Professional Affiliation | | |
| Academic | 6 | 12 |
| Government | 3 | 0 |
| Research Role | | |
| Principal Investigator | 6 | 11 |
| Co-Investigator | 8 | 7 |
| Research Coordinator | 2 | 0 |
| Data Coordinator | 2 | 0 |
| Operational Manager | 1 | 0 |
| Research Assistant | 1 | 0 |
| Career Stage | | |
| Early Career | 2 | 2 |
| Mid-Career | 5 | 6 |
| Late Career | 2 | 4 |
| Area of HIV Research | | |
| Epidemiology | 6 | 8 |
| Social/Behavioral | 6 | 8 |
| Prevention | 5 | 4 |
| MTCT | 2 | 3 |
| Co-Infections | 3 | 6 |
| Implementation | 3 | 8 |
| Community Engagement | 1 | 6 |
| HIV Cure/Vaccine | 0 | 1 |
| Other | 2 | 0 |
| Primary Country of Affiliation | South Africa (n=2)  Nigeria (n=4)  Uganda (n=1)  Kenya (n=1) | United States (n=11)  United Kingdom (n=1) |

Completion was not required and not all participants completed the demographics section of the survey, resulting in different response numbers between the demographic information collected and overall survey responses.
